# Regulator of G-Protein Signaling (RGS) Protein Modulation of Opioid Receptor Signaling as a Potential Target for Pain Management

**DOI:** 10.3389/fnmol.2020.00005

**Published:** 2020-01-24

**Authors:** Nicolas B. Senese, Ram Kandasamy, Kelsey E. Kochan, John R. Traynor

**Affiliations:** ^1^Department of Pharmacology, Edward F. Domino Research Center, University of Michigan Medical School, Ann Arbor, MI, United States; ^2^Department of Psychiatry, Chicago, IL, United States; ^3^Department of Psychology, California State University, East Bay, Hayward, CA, United States; ^4^Department of Medicinal Chemistry, College of Pharmacy, University of Michigan, Ann Arbor, MI, United States

**Keywords:** analgesia, G-proteins, opioid receptors, pain, RGS proteins, signaling

## Abstract

Opioid drugs are the gold standard for the management of pain, but their use is severely limited by dangerous and unpleasant side effects. All clinically available opioid analgesics bind to and activate the mu-opioid receptor (MOR), a heterotrimeric G-protein-coupled receptor, to produce analgesia. The activity of these receptors is modulated by a family of intracellular RGS proteins or regulators of G-protein signaling proteins, characterized by the presence of a conserved RGS Homology (RH) domain. These proteins act as negative regulators of G-protein signaling by serving as GTPase accelerating proteins or GAPS to switch off signaling by both the Gα and βγ subunits of heterotrimeric G-proteins. Consequently, knockdown or knockout of RGS protein activity enhances signaling downstream of MOR. In this review we discuss current knowledge of how this activity, across the different families of RGS proteins, modulates MOR activity, as well as activity of other members of the opioid receptor family, and so pain and analgesia in animal models, with particular emphasis on RGS4 and RGS9 families. We discuss inhibition of RGS proteins with small molecule inhibitors that bind to sensitive cysteine moieties in the RH domain and the potential for targeting this family of intracellular proteins as adjuncts to provide an opioid sparing effect or as standalone analgesics by promoting the activity of endogenous opioid peptides. Overall, we conclude that RGS proteins may be a novel drug target to provide analgesia with reduced opioid-like side effects, but that much basic work is needed to define the roles for specific RGS proteins, particularly in chronic pain, as well as a need to develop newer inhibitors.

## Introduction

Pain is a significant problem worldwide, and adequate pain relief remains an unmet medical need. Opioids acting at the mu-opioid receptor (MOR), a G-protein coupled receptor (GPCR), have been used therapeutically to control pain for centuries and remain the most commonly used class of analgesics and the most effective option for many patients. This, along with an increased focus on completely eliminating pain among physicians, has led to the recent huge increase in opioid prescriptions which, together with the addiction liability and respiratory depressant properties of opioid drugs, has driven the current opioid crisis and the resultant dramatic increase in opioid overdose deaths (Babu et al., 2019). Nonetheless, opioids remain the gold standard for pain control. Consequently, many approaches are being taken to target MOR in ways that enhance analgesic properties but reduce unwanted effects including, allosteric modulators (Burford et al., 2013), biased agonists that preferentially stimulate certain downstream pathways (Manglik et al., 2016; Schmid et al., 2017), compounds that target several opioid receptors simultaneously (Nastase et al., 2018) or compounds with slow access to central MORs (Markman et al., 2019). In this review we discuss ways in which intracellular processes downstream of MOR activation by both exogenous opioid drugs and endogenous opioid peptides can be manipulated by regulator of G-protein signaling (RGS) proteins, and if this provides an avenue for the development of new analgesic molecules.

## RGS Proteins

**FIGURE 1:**
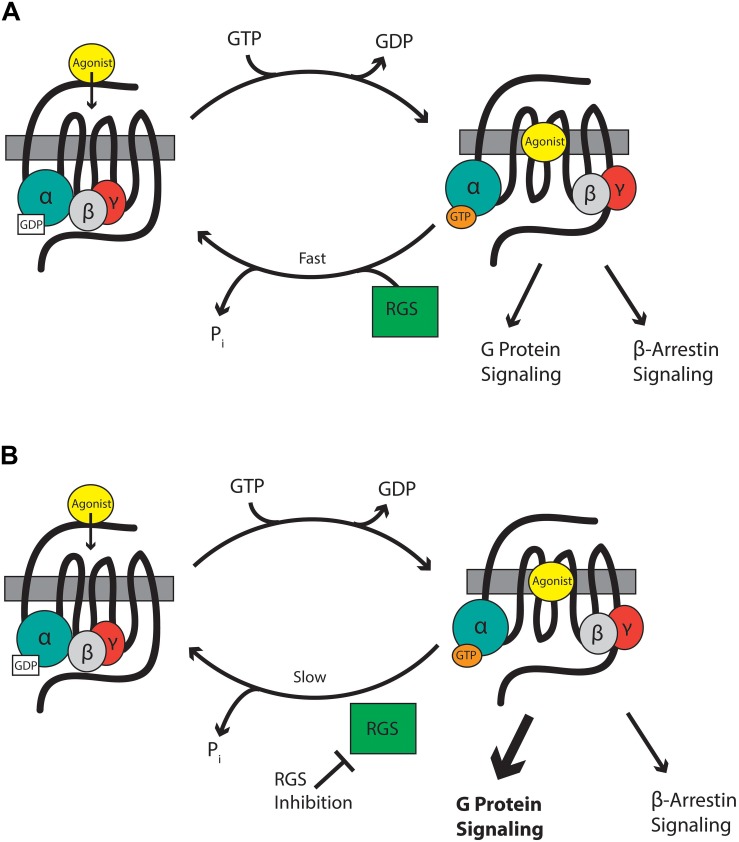
**(A)** RGS proteins accelerate the hydrolysis of GTP bound to the Gα subunit to terminate signaling by reformation of the G-protein heterodimer. **(B)** RGS inhibitors slow the hydrolysis of GTP and so enhance signaling through Gα and βγ proteins.

Mu-opioid receptors are seven-transmembrane domain GPCRs that interact with G-proteins of the Gα_i/o_ and Gα_z_ classes that form a heterotrimer with their essential β and γ subunits (Figure 1). At rest, the Gαβγ heterotrimer is bound to GDP. GPCR activation leads to dissociation of GDP from the Gα subunit and its replacement with GTP causing the Gα-bound GTP to separate from the βγ heterodimer. The now active Gα-GTP and βγ subunits interact with intracellular signaling partners, including inwardly rectifying potassium channels, calcium channels, phospholipase C, adenylyl cyclase isoforms, and components of the mitogen-activated protein kinase (MAPK) pathway. Intracellular signaling is terminated when endogenous GTPase activity of Gα hydrolyses GTP to GDP. The formed Gα-GDP then reassociates with the βγ heterodimer to terminate signaling. The enzymatic GTPase activity of the Gα_i/o/z_ subunits is slow with a GTP turnover rate of 2–5 per minute. This is not fast enough to allow a cell to respond to subsequent incoming signals. Here, RGS proteins come into play. These proteins bind to the switch regions of the active, GTP-bound Gα (Tesmer et al., 1997) and act as GTPase accelerating proteins or GAPs to increase rate of GTP hydrolysis by up to 100-fold. This drastically shortens the lifetime of the active Gα-GTP and βγ signaling proteins, resulting in a negative regulation of GPCR signaling, including signaling downstream of MOR (Figure 1).

The RGS proteins themselves constitute a 20-plus member family of intracellular regulatory proteins characterized by an RGS-homology (RH) domain and divided into subfamilies according to domain- and sequence-homology (Hollinger and Hepler, 2002). RGS proteins vary in size and complexity from simple N- and C-terminal extensions to more complex proteins (Table 1). Some members of the family are selective for certain G-protein subtypes (Posner et al., 1999; Lan et al., 2000) and receptors (Xu et al., 1999; Wang et al., 2009). RGS proteins are variously expressed throughout the body including pain pathways in the central nervous system (CNS) where expression overlaps with MOR expression, particularly for RGS4 and the splice variant of RGS9, RGS9-2 (Gold et al., 1997; Peckys and Landwehrmeyer, 1999; Grafstein-Dunn et al., 2001; Traynor and Neubig, 2005). For example, the small RGS4 protein is expressed in many structures involved in the transmission and maintenance of pain, including the dorsal horn of the spinal cord, the periaqueductal gray (PAG), the thalamus, and the basal ganglia (Ni et al., 1999; Gold et al., 2003; Terzi et al., 2009; Taccola et al., 2016).

**TABLE 1:** RGS subfamily characterization and expression.

**RGS family**	**Domains present**	**Name**	**G-protein specificity**	**CNS expression**
RZ	Cysteine-string	RGS17 (RGSZ2)	Gi, Gz, Gq	Isocortex, OLF, HPF, CTXsp, PAL, TH, HY, MB, P, MY
		RGS19 (GAIP)	Gi, Gz, Gq	Isocortex, OLF, HPF, CTXsp, STR, PAL, TH, MB, P, MY, CB
		RGS20 (RGSZ1)	Gi, Gz	Isocortex, CTXsp, STR, PAL
R4	N-terminal amphipathic sequence	RGS1	Gi, Gq	N/A
		RGS2	Gi < Gq	CTXsp, STR, PAL
		RGS3	Gi, Gq	TH, CB
		RGS4	Gi, Gq	Isocortex, OLF, HPF, CTXsp, STR, PAL, TH, HY, MB, P, MY, CB
		RGS5	Gi, Gq	Isocortex, OLF, HPF, CTXsp, STR, PAL, TH, HY, MB, P, MY, CB
		RGS8	Gi, Gq	Isocortex, OLF, HPF, CTXsp, STR, PAL, TH, HY, MB, P, MY, CB
		RGS13	N/A	N/A
		RGS16	Gi, Gq	TH
		RGS18	Gi, Gq	N/A
		RGS21	Gi, Gq	N/A
R7	GGL-(Gβ5) DEP-(R9AP, R7BP)	RGS6	Go	HPF, CTX (Ahlers et al., 2016)
		RGS7	Go > Gi2 > Gi1	Isocortex, OLF, HPF, CTXsp, STR, PAL, TH, HY, MB, P, MY, CB
		RGS9	Go	Isocortex, OLF, CTXsp, STR, PAL, HY
		RGS11	Go	Isocortex, OLF, HPF, CTXsp, STR, PAL, TH, HY, MB, P, MY
R12	GoLoco-(Gα-GDP) RBD-(rap) PDZ	RGS10	Gi	Isocortex, OLF, HPF, CTXsp, STR, PAL, TH, HY, MB, P, MY, CB
		RGS12	Gi	Isocortex, OLF, HPF, CTXsp, STR, PAL, TH, HY, MB, P, MY, CB
		RGS14	Gi	Isocortex, OLF, HPF, CTXsp, STR

*List of RGS families and their respective domains, G-protein specificities, and expression in the mouse central nervous system. Isocortex, isocortex; OLF, olfactory; HPF, hippocampal formation; CTXsp, cortical subplate; STR, striatum; PAL, pallidum; TH, thalamus; HY, hypothalamus; MB, midbrain; P, pons; MY, medulla; CB, cerebellum. N/A = data not available. Taken from the Allen Brain Atlas (https://mouse.brain-map.org), except where stated.*

## Rationale for RGS Proteins as Potential Targets for Pain Management

Intracellular proteins are not usually considered suitable drug targets due to their ubiquitous expression. In contrast, their differential expression patterns, selectivity for specific receptors and specificity for particular G-proteins, although not absolute, suggests the possibility that RGS proteins could be attractive drug targets for the management of pain by enhancing MOR-mediated signaling, leading to enhanced antinociception. Drugs inhibiting RGS activity could be beneficial in several ways. First, an enhancement of action of morphine and related exogenous opioid drugs would result in an opioid sparing effect, which would be especially advantageous if different RGS proteins controlled MOR signaling in those neuronal systems leading to antinociception versus those responsible for side-effects of respiration, reward, and constipation. Second, RGS inhibitors could produce analgesia in their own right by enhancing endogenous opioid peptide activity even in the absence of exogenous opioid drugs. Opioid peptides are released at spinal and supraspinal sites during pain (Levine et al., 1978; Cesselin et al., 1980; Porro et al., 1991; Zangen et al., 1998; Hurley and Hammond, 2001; Wu et al., 2001) and also at peripheral sites (Stein et al., 2003). These endogenous peptides offer limited protection against pain but this effect is significantly increased if enzymatic peptide breakdown is prevented by so-called “enkephalinase inhibitors” (Fournié-Zaluski et al., 1992; Noble et al., 1992, 1997). Enhancement of endogenous opioid peptide signaling downstream of MOR by inhibition of RGS action should increase the analgesic efficacy of the peptides. Moreover, this approach has the advantage that, unlike enkephalinase inhibitors which globally increase enkephalin levels, the spatial and temporal release of the peptides would be retained and so RGS inhibitors will be effective only in those areas where the peptides are released in response to noxious stimuli, but not in areas responsible other actions of the peptides. A similar concept has recently been discussed with regard to positive allosteric modulators of MOR (Burford et al., 2015; Livingston and Traynor, 2018). Thirdly, there is evidence that the beneficial analgesic action of MOR agonists (and the endogenous opioid peptides) is due to signaling downstream of G-proteins, whereas the unwanted effects of respiratory depression and constipation may be mediated via the β-arrestin pathway (Raehal et al., 2011; Violin et al., 2014; Manglik et al., 2016; Schmid et al., 2017). Since RGS proteins modulate the G-protein component of MOR signaling, but not the β-arrestin component, inhibitors of RGS proteins would be expected to show an increased therapeutic window separating the beneficial from unwanted effects (Figure 1).

On the other hand, while RGS proteins are attractive analgesic targets and there is some degree of selectivity of expression and interaction with opioid receptors and G-proteins inhibition of RGS activity could regulate signaling downstream of numerous GPCRs. This suggests more nuanced strategies may be required to avoid the potential for off-target effects. Such targets might be the interface between RGS and opioid receptors or the complete RGS-Gα-opioid receptor complex rather than the RGS protein in isolation avoiding the potential for off-target effects.

## RGS Insensitive Gα-Proteins

Because RGS proteins constitute a large family of molecules it is difficult to know where to start when assessing their ability to control MOR signaling. An easier way is to develop a system which genetically knocks out all RGS GAP activity. This is feasible since replacement of a Gly in the “switch 3” region of the Gα protein (Tesmer et al., 1997) with Ser blocks the interaction between the RH region of RGS protein and the GTP-bound Gα subunit, without affecting any other properties of the Gα protein including GDP release, GTP hydrolysis, Gβγ binding, or interaction with the receptor. This mutation therefore prevents all GAP activity at a specific Gα protein. Thus, for example the Gly-Ser mutation in Gα_o_, promotes signaling downstream of MOR *in vitro* (Clark et al., 2003).

The behavioral effects of the mutation can be studied in mice with knock-in of RGS-insensitive Gα proteins (RGSi-Gα). This allows for proof of principle that inhibition of RGS activity is a viable strategy to provide antinociception and avoids the possibility of redundancy of GAP activity. Although on the minus side this approach does not identify the specific RGS protein(s) involved.

In assays using heat as the nociceptive source, mice expressing RGSi-Gα_o_ displayed an enhanced baseline withdrawal latency that was reversed by naltrexone, showing that endogenous opioid peptide activity is increased when RGS action is nullified (Lamberts et al., 2011). Similarly in the hot-plate test morphine-induced antinociception was enhanced; these finding were supported by an increased opioid-peptide mediated disinhibition of GABA release in the PAG, an important region for descending pain control (Lamberts et al., 2013). Surprisingly, in the tail withdrawal test the action of morphine was decreased, suggesting a permissive, not inhibitory, action for RGS proteins. Indeed, in the PAG, MOR activation of G-protein-gated inwardly rectifying potassium channels (GIRKs) was reduced for morphine and fentanyl in mice expressing RGSi-Gα_o_ proteins. No effect was seen on methionine enkephalin modulation of GIRK currents because this endogenous ligand appeared to use Gα_*i*_ proteins which are still regulated by RGS proteins in this genetic model (McPherson et al., 2018). The results indicate that the RGS-mediated reduction in opioid-induced GIRK activation in mice expressing RGSi-Gα_o_ plays a role in opioid spinal antinociception, but not supraspinal, antinociception. These studies indicate that in general RGS protein GAP activity can produce negative and positive regulation of signaling depending on the intracellular effector(s) involved. One mechanism for this is RGS-mediated “kinetic scaffolding,” the results of which depend on the proximity of the various components within a cell (Zhong et al., 2003). In this model when effectors are close to the receptor RGS proteins are permissive because they act to sustain local concentrations of Gα-GDP necessary to maintain G-protein signaling. In contrast, further from the receptor where Gα-GDP is not depleted, RGS proteins suppress signaling and so are inhibitory. Alternatively, the opposite responses in morphine pharmacology observed could be due to roles for RGS proteins that have complex, for example, scaffolding functions. Additionally, since different neuronal circuits are involved in the two measures of morphine antinociception the loss of RGS negative regulation of Gα_o_ could reveal constitutive activity of opposing transmitted systems that use this G-protein, for example the nociceptin peptide system (Bertorelli et al., 1999; Khroyan et al., 2009).

## Specific Families of RGS Proteins

While use of RGS-insensitive Gα_o_ proteins can provide proof of principle, conflicting results, such as in the antinociceptive assays discussed above highlight drawbacks in this approach. As such, examination of individual RGS proteins is needed to identify discrete pharmacological targets.

### R4 Family

RGS4 itself has been extensively studied with respect to opioid-mediated signaling and antinociception This protein is distributed widely throughout the CNS where it regulates the pharmacology of MOR agonists (Table 1, reviewed in Traynor and Neubig, 2005). RGS4 is thought to interact directly with MOR *via* the fourth intracellular loop of the receptor (residues 329–355) and the RGS4 N-terminal domain (Leontiadis et al., 2009). Removal of the N-terminal domain not only reduces RGS4-receptor interactions, but eliminates the receptor selectivity of endogenous RGS4 protein (Zeng et al., 1998; Leontiadis et al., 2009). When overexpressed in HEK293 cells, RGS4 is localized throughout the cytosol, nucleus, and plasma membrane (Roy et al., 2003) and binds only weakly to Gα_i/o_ proteins. However, following application of the MOR agonist [D-Ala^2^, N-MePhe^4^, Gly-ol]-enkephalin (DAMGO), expression shifts to the plasma membrane such that RGS4 is co-localized with the receptor (Leontiadis et al., 2009) and the interaction between the two proteins is enhanced. In contrast, in SH-SY5Y human neuroblastoma cells that endogenously express RGS4 and MOR, knockdown of RGS4 did not affect responses to the MOR agonist morphine (Wang et al., 2009), suggesting that the ability of RGS4 to regulate MOR may be determined by the cell type and/or the agonist.

Intracerebroventricular (i.c.v.) administration in male mice of antisense-DNA against RGS4 resulted in a greater response to i.c.v. morphine in the tail withdrawal test compared to control given scrambled antisense (Garzón et al., 2001). In contrast, constitutive RGS4 knockout mice do not display alterations in pain sensitivity in tests of acute nociception (Grillet et al., 2005). This finding may be due to redundancy of RGS action. That is, since RGS4 is essentially an RH domain with very short N and C-termini, its loss may easily be compensated for by other RGS proteins (Doupnik, 2015), or by physiological compensatory mechanisms also regulated by RGS4. Indeed, further studies have implicated a critical role for RGS4 in the nucleus accumbens (NAc) in opioid antinociception (Han et al., 2010). Conditional knockout of RGS4 only in this brain region reduces fentanyl and methadone antinociception, but not that of morphine, although it does act as a negative modulator of the rewarding effects of morphine, suggesting both agonist-specific and tissue-specific outcomes (Han et al., 2010). To explain this discrepancy, the authors used immunoprecipitation experiments to indicate that fentanyl, but not morphine, recruits Gα_q_, rather than Gα_i/o_ proteins to MOR in the NAc and this competes with RGS4 for association with the receptor (Han et al., 2010).

**FIGURE 2:**
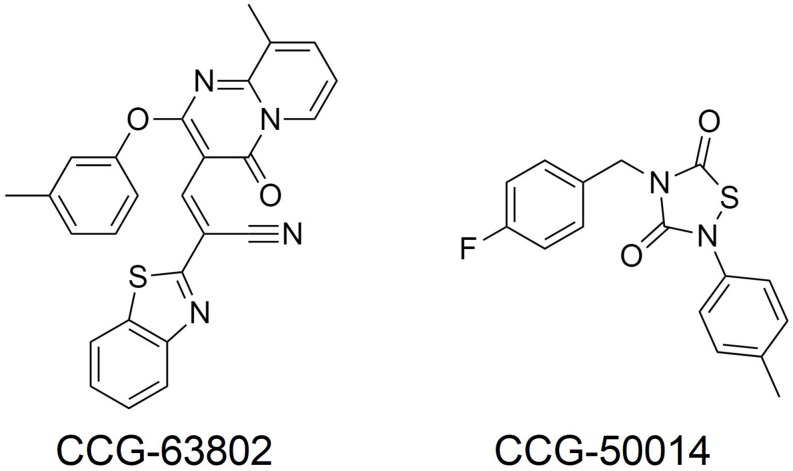
Small molecule RGS inhibitors used in preclinical analgesic studies.

A more important role for RGS4 might be in chronic pain states, where RGS4 expression is dynamically regulated. For example, following sciatic nerve injury in the rat there is an up-regulation of RGS4 mRNA expression in the dorsal horn of the spinal cord, with no change in the mRNA of other RGS proteins measured (RGS6, 7, 8, 9, 11, 12, 14, 17, 19; Garnier et al., 2003; Bosier et al., 2015; Taccola et al., 2016). At the same time rats become hypersensitive to noxious stimuli and the potency of MOR agonists decreases. Chronic pain states, such as following sciatic nerve injury are less sensitive to control by opioids than acute pain. Since RGS4 negatively regulates MOR signaling *in vitro* and there is significant overlap in expression of RGS4 and MOR within the spinal dorsal horn (Peckys and Landwehrmeyer, 1999; Garnier et al., 2003), this increased expression of RGS4 likely contributes to the loss of morphine potency in chronic pain. A report also indicates increases in RGS3 mRNA in the dorsal horn of the lumbar spinal cord after sciatic nerve ligation, although RGS4 levels decrease several days later; these effects may involve astrocyte RGS proteins (Doyen et al., 2017). In support of a role for up-regulated RGS4 (and RGS3) in reducing the effectiveness of morphine, use of the inhibitor CCG-63802 (Figure 2; Blazer et al., 2010) to prevent RGS4 action attenuates hyperalgesia following nerve injury (Bosier et al., 2015; Taccola et al., 2016). This attenuation can be attributed to the rescue of tonically active endogenous antinociception systems, such as the enkephalins, although in one study using this inhibitor (Bosier et al., 2015) endogenous cannabinoids rather than opioid endogenous opioids were implicated.

In contrast to changes in the spinal cord, RGS4 (as well as RGS3) message which is high in C-fiber sensory neurons in dorsal root ganglia (DRG) of the rat, has been reported to decrease following transection of the sciatic nerve (Costigan et al., 2003), suggesting a location-dependent regulation of this transcript. A reduction in RGS4 would lead to an increase in GPCR, possibly MOR signaling. These divergent effects of up- or down-regulation of RGS proteins indicate a very fine level of pathophysiological control, although of course we do not know the specific GPCR target or targets of either the up-regulated RGS4/RGS3 in the dorsal horn or the down regulated RGS4 in the DRG. Resolving the reasons for the different findings on RGS3/4 levels after sciatic nerve injury and identifying the GPCRs that are modulated by these proteins would be an important step forward in developing RGS protein-based analgesics or analgesic adjuncts.

Injection of formalin into the mouse hind paw produces a biphasic hyperalgesia consisting of an early phase and a late phase. Mice lacking RGS4 are less hyperalgesic during the late phase (Yoon et al., 2015; Avrampou et al., 2019). Moreover, mice lacking RGS4 recover more quickly from mechanical and cold hypersensitivity following inflammation, caused by Freund’s Complete Adjuvant (CFA) injection into the hind paw, or nerve injury and show recovery of wheel running as a measure of pain-depressed behavior (Avrampou et al., 2019). One potential mechanism to explain both of these observations is the loss of negative regulation by RGS4 of endogenous antinociceptive signaling, possibly involving opioid peptides released in response to the persistent inflammatory pain (Porro et al., 1991; Zangen et al., 1998; Wu et al., 2001). As with the nerve injury studies using CCG-63802 discussed above, results from this experimental paradigm support the notion that an inhibitor of RGS4 should enhance endogenous pain systems to produce analgesia, even in the absence of opioid drugs such as morphine. In support of this concept, the potency of the stable enkephalin analog DAMGO was increased 10-fold in the formalin test in animals lacking RGS4 compared to their wild-type controls (Yoon et al., 2015), and intrathecal (i.t.) administration of the small molecule RGS4 inhibitor CCG-50014 (Figure 2; Blazer et al., 2011) to wild-type mice produced dose-dependent antinociception on its own that was blocked by the opioid antagonist naloxone, as well as enhancing the action of DAMGO (Yoon et al., 2015).

Chronic pain is a highly complex condition. Using conditional knockdown of RGS4 Avrampou et al. (2019) demonstrated that a major contributor to reversal of cold and mechanical hypersensitivity, but not recovery of pain-depressed wheel running, is the ventral posterolateral region of the thalamus, an important center for relaying nociceptive information. RNA sequence analysis of thalamic tissue from wild-type and RGS4 knockout mice after CFA-induced inflammatory pain showed decreased changes in gene expression in the knockout group and identified differences that included components of glutamatergic signaling, including an increased expression of membrane bound metabotropic glutamate receptor 2 which has been associated with recovery from hypersensitivity to sensory stimuli. Finally, the role for RGS4 in the anti-allodynic actions of tricyclic antidepressants and the NMDA receptor antagonist ketamine have been studied following sciatic nerve injury (Stratinaki et al., 2013). The antiallodynic action of chronic low dose, but not high dose, desipramine was reduced in RGS4 knockout mice, whereas a low dose of ketamine produced antiallodynic behavior only in mice lacking RGS4. This difference highlights the complex roles of RGS4 and the fact that the GPCR targets for RGS4 have to be carefully considered when proposing inhibitors of RGS4 for the management of chronic pain. Nonetheless, whatever the mechanism involved in chronic pain and the drugs used for its management, these studies do suggest it is worth further investigating inhibitors of RGS4 as standalone treatments for chronic pain. The fact that two independently generated RGS4 knockout mouse lines, with distinct genetic backgrounds, show no overt behavioral abnormalities (Grillet et al., 2003; Han et al., 2010; Avrampou et al., 2019) provides support for RGS4 as a therapeutic target for pain management.

Roles for other R4 family members in modulating opioid function and analgesia have not been explored so extensively. RGS8 is enriched in the thalamus (Gold et al., 1997), a region dense in MOR expression, and RGS8 acts as a GAP for opioid-mediated signaling (Talbot et al., 2010). Thus, it is possible that RGS8 interacts with MORs to modulate signaling in the thalamus to regulate processing of nociceptive information. Knockdown of RGS2 and RGS3 was reported to have no effect on baseline antinociception in the tail-flick test, but to inhibit the antinociceptive response to morphine and the endogenous opioid β-endorphin (Garzón et al., 2001), suggesting, a positive role for these proteins in opioid antinociception. In contrast, after RGS16 knockdown, mice showed an increased antinociceptive response to morphine (Garzón et al., 2001). The basis of these opposing effects of different R4 family members has not been adequately explored, although it has been suggested that distinct Gα interaction profiles between the different RGS proteins may be responsible for the diverse effects (Garzón et al., 2000, 2001).

Further studies of other RGS4 family members in both acute and chronic pain states are warranted. In particular, there are no published studies of family members other than RGS3 and 4 in chronic pain.

### R7 Family

The R7 family of RGS proteins comprises RGS6, 7, 9–1, 9–2, and 11 (Hollinger and Hepler, 2002). RGS9-1 and -2 are variants that differ only in the C-terminal tail. RGS9-1 is found only in the retina whereas RGS9-2 is brain specific, and highly expressed in the striatum. RGS7 and RGS9-2 form a heterodimer with the type 5 G-protein β (Gβ5) subunit. This facilitates correct folding and provides proteolytic stability. In addition, both RGS7/Gβ5 and RGS9-2/Gβ5 form complexes with a small palmitoylated protein R7 Binding Protein (R7BP), to control membrane localization and stability (reviewed in Lamberts and Traynor, 2013).

In permeabilized C6 glioma cells expressing MOR and Gα_i__2_, addition of the RH region of RGS7 did not affect DAMGO-induced inhibition of cAMP accumulation (Talbot et al., 2010). When Gα_o_ was expressed instead of Gα_i__2_, addition of the RH domain of RGS7 effectively inhibited the actions of DAMGO (Talbot et al., 2010), suggesting that RGS7 selectively regulates the action of MOR depending on the G-protein expressed. This selectivity may be due to a lack of physical interaction between RGS7 and Gα_i__2_, as increasing concentrations of Gα_i__2_ disrupted the RGS4/Gα_o_ complex but not the RGS7/Gα_o_ complex (Talbot et al., 2010). This suggests that the inability of RGS7 to regulate MOR signaling in cells expressing Gα_i__2_ is due to a failure of RGS7/Gα_i__2_ complex formation.

RGS9-2 interacts with MOR to prevent several events triggered by receptor activation. When RGS9-2 is located close to the cell membrane, it delays agonist-induced internalization of MOR (Psifogeorgou et al., 2007). Further, morphine promotes the association of RGS9-2 with β-arrestin-2, a key component of MOR desensitization (Psifogeorgou et al., 2007). This association is dampened in the presence of the structurally different MOR agonist fentanyl (Psifogeorgou et al., 2011). Thus, RGS9-2 plays an important role in MOR regulation, as it negatively regulates signaling downstream of MOR and inhibits receptor endocytosis; however, these effects appear to be agonist-dependent.

Male mice given antisense-DNA against RGS7 or RGS9-2 into the ventricles, showed greater responses to morphine, DAMGO, and β-endorphin in the tail-flick test (Garzón et al., 2001; Sánchez-Blázquez et al., 2003). Knockdown of RGS9-2 or RGS11 enhanced DAMGO antinociception to a greater degree than knockdown of RGS6 or RGS7 (Garzón et al., 2003). The antinociceptive action of morphine toward an acute heat stimulus was reported to be enhanced in mice completely lacking RGS9 (Zachariou et al., 2003; Papachatzaki et al., 2011). The effects again appear to depend on the agonist studied. Thus, in the same RGS9 knockout mice, the analgesic efficacy of oxycodone was not changed, in either acute pain or in sciatic nerve injury induced pain (Gaspari et al., 2017) and whereas RGS9-2 knockout enhances the action of morphine in the hot-plate test, there is an inhibition of fentanyl- and methadone-mediated antinociception (Papachatzaki et al., 2011). This differential behavior across agonists that bind to the same orthosteric site on the MOR has been explained by the formation of dissimilar protein complexes following binding of ligand to MOR. In other words, there is a biased activation of receptor such that morphine promotes an association between RGS9-2 and Gα_i__3_ whereas RGS9-2/Gα_*q*_ complexes are seen with the other ligands.

The opening of inwardly rectifying potassium (GIRK) channels is an important mechanism for antinociception downstream of MOR. Such channels are modulated by a complex of RGS 7 or 9 with Gβ5 downstream of MOR that is allosterically controlled by R7BP. Thus, as might be expected the loss of other components of this complex also result in altered antinociception. In the absence of R7BP there is a loss of negative regulation of MOR signaling, enhancing GIRK activity and so increasing morphine- and fentanyl induced antinociception (Terzi et al., 2012; Zhou et al., 2012). The R7BP null mice also show an enhanced basal latency to an acute thermal stimulus in the hot-plate assay (Zhou et al., 2012), indicating enhanced endogenous antinociception by an increase in the activity of endogenous opioid peptides acting at MOR (Lamberts et al., 2011).

In support of an enhancement of endogenous opioid antinociception, RGS9 knockout mice exhibit a small degree of reduced hypersensitivity to the sensory component of both thermal and mechanical insult in early stages of neuropathic pain but exacerbation of affective components of the pain at later time points (Terzi et al., 2014). The nerve injury in wild-type mice was seen to cause a transient reduction in levels of RGS9-2 in the spinal cord, explaining the reduced sensory hypersensitivity, although phenotypic changes in basal antinociceptive activity have not been ruled out, and a later decrease in RGS9-2 levels in the NAc, explaining the change in the affective response. Consequently, RGS9-2 appears to be a negative regulator of the sensory component but a positive regulator of the affective response (Terzi et al., 2014). Since neuropathic pain can lead to depression in humans (Kroenke et al., 2009) this complication might preclude the use of inhibitors of RGS9-2 in the management of chronic pain.

### R12 Family

The R12 family of RGS proteins consists of RGS10, 12, and 14. Little is known about how the R12 RGS family regulates MOR signaling and/or analgesia. Central knockdown of RGS12 and RGS14 increased morphine antinociception on the tail-flick test in the mouse, although there were no reported changes in baseline nociceptive thresholds (Garzón et al., 2001) which may suggest a low or absent release of endogenous peptides or a lack of co-localization with RGS proteins. RGS14 knockdown reduced the development of acute tolerance following morphine exposure, and these behavioral changes occurred alongside increased MOR phosphorylation, which promotes internalization and recycling of the receptor (Rodríguez-Muñoz et al., 2007a). This suggests that in normal circumstances RGS14 limits agonist activity in a way that reduces both MOR phosphorylation (e.g., by GRKs) and β-arrestin-mediated endocytosis, leading to more robust receptor desensitization than in systems lacking RGS14 (Rodríguez-Muñoz et al., 2007a). More work is needed to understand the potential roles for the R12 family in regulating MOR signaling and antinociception.

### RZ Family

The RZ family of RGS proteins consists of RGS17 (also known as RGSZ2), 19 (also known as G Alpha Interacting Protein/GAIP), and RGS20 (RGSZ1). Antisense knockdown of central RGSZ17 levels in male mice was seen to increase morphine and DAMGO antinociception, but also increased the rate of tolerance development (Garzón et al., 2005). Knockdown of RGS19 in SH-SY5Y cells enhances MOR agonist-induced MAPK stimulation and adenylyl cyclase inhibition (Wang and Traynor, 2013). Consequently, knockdown of RGS19 and RGS20, enhances the antinociceptive effects of morphine and DAMGO (Garzón et al., 2004). In addition to these effects on antinociception, knockdown of either RGS19 or RGS20 increased the rate of analgesic tolerance development (Garzón et al., 2004). Suppressing RGS20 function increased the antinociceptive efficacy of MOR agonists and delayed the development of morphine tolerance in mice (Gaspari et al., 2018). Thus, both RGS20 (RGSZ1) and RGS17 (RGSZ2) appear to play roles in regulating opioid antinociception and tolerance.

Treatment with morphine or DAMGO decreases associations between MOR and Gα_i__2_ but increases associations between Gα_i__2_ and RGSZ2 (Rodríguez-Muñoz et al., 2007b). This shift is transient, and the time course mimics the duration of antinociceptive tolerance following acute administration of morphine, such that Gα_i__2_ interactions have returned to normal at time points when acute antinociceptive tolerance has waned (Rodríguez-Muñoz et al., 2007b). A similar process occurs with RGSZ2 and Gα_z_, with MOR agonists increasing association between these proteins while decreasing Gα_z_/MOR association (Garzón et al., 2005). Together these results suggest that all RZ RGS proteins are capable of both inhibiting MOR agonist-induced antinociception and reducing the development of tolerance following agonist exposure, likely through regulation of Gα_z_ and Gα_i__2_ (Garzón et al., 2004, 2005; Rodríguez-Muñoz et al., 2007b).

To date, no studies related to antinociception have been performed in mice with constitutive or conditional genetic knockout of any members of the RGS RZ family.

## RGS Regulation of Signaling and Antinociception Downstream of Other Opioid Receptors

All members of the opioid receptor family are involved in some way in the modulation of pain and have been the subject of study in relation to their interactions with RGS proteins.

### The Delta Opioid Receptor (DOR)

There is much evidence that RGS4 modulates signaling downstream of the delta opioid receptor (DOR) and this in turn leads to increases in antinociceptive properties of agonists at these receptors. For example, purified RGS4 reverses the enkephalin-mediated DOR inhibition of adenylate cyclase activity in NG108-15 cells (Hepler et al., 1997). In HEK293 cells, RGS4 overexpression similarly reduced DOR agonist-stimulated signaling and increased the degree of DOR internalization (Leontiadis et al., 2009). In agreement with these overexpression studies, a 90% reduction of RGS4 in SH-SY5Y cells significantly increased the ability of DOR agonists to inhibit adenylyl cyclase and activate MAPK (Wang et al., 2009) and in mouse brain the small molecule DOR agonist SNC80 increased striatal MAPK phosphorylation to a greater degree in RGS4 knockout animals, than their littermate controls (Dripps et al., 2017). Mutagenesis studies have identified the C-terminus of DOR as the site of interaction with RGS4 and work using molecular dynamics simulations and *in vitro* pull-down experiments, has isolated this to 12 amino-acid residues in helix 8 of DOR and to the first 17 N-terminal residues of RGS4 (Karoussiotis et al., 2019).

Similar to the enhancement of MOR-mediated antinociception, the potency of SNC80 is increased in nitroglycerin-induced hyperalgesia in mice expressing RGSi-Gα_o_ (Dripps et al., 2017). In addition, RGS4 knockout mice show an enhanced antinociceptive response to SNC80 compared to their wild-type littermate controls (Dripps et al., 2017). Importantly, in both the RGSi-Gα_o_ knock-in mice and the RGS4 knockout mice the enhancement of DOR-mediated antinociception occurs without an increased ability of SNC80 to cause convulsions, a serious side effect of DOR agonists, thus increasing the preclinical therapeutic window of this DOR agonists.

RGS19 (GAIP) has also been studied with respect to DOR signaling. Purified RGS19, like RGS4 acts as a GAP for DOR signaling in NG108-15 cells (Hepler et al., 1997). In contrast DOR signaling in SH-SY5Y cells to adenylate cyclase or MAPK was not sensitive to knockdown of RGS19 (Wang and Traynor, 2013) and knockdown of RGS19 failed to modulate antinociceptive responses to DOR agonists, DPDPE and deltorphin (Garzón et al., 2004). This could suggest the experiments in NG108-15 cells that RGS19 is acting as a non-selective GAP. Conversely, there is other evidence of a role for RGS19 in DOR signaling. Thus, in HEK cells Flag-tagged DOR and heterologously expressed RGS19 are found in different cellular compartments; RGS19 in clathrin-coated membrane regions and DOR near Gα_i__3_ in non-clathrin-coated regions (Elenko et al., 2003). Following DOR agonist treatment, activated GTP-bound Gα_i__3_ and RGS19 co-localize in clathrin-coated regions to form a complex when RGS19 acts as a GAP to promote GTP hydrolysis returning Gα_i__3_ to its GDP bound inactive form. This is reminiscent of the proposed process, described above, where MOR agonist treatment shifts Gα_i__2_ and Gα_z_ from a complex with MOR to a complex with RGSZ-2 (Rodríguez-Muñoz et al., 2007b).

### The Kappa Opioid Receptor

Little published work is available on how RGS proteins affect signaling and antinociception downstream of the kappa opioid receptor, KOR. The genetic loci for RGS20 and KOR are separated by only approximately 600 base pairs, suggesting that these proteins may be co-regulated (Sierra et al., 2002). In *Xenopus* oocytes, RGS4 expression inhibits GIRK1 and GIRK2 downstream of KOR activation, and the presence of RGS4 appears to counteract cellular adaptations to sustained KOR agonist treatment (Ulens et al., 2000). Further, RGS2 and RGS4 bind to different domains of KOR to reduce signaling of this receptor to adenylate cyclase and the MAP kinase pathway in HEK cells (Papakonstantinou et al., 2015). In PC12 cells stably expressing KOR, agonist application increased RGS4 mRNA expression in a KOR antagonist reversible manner, a process that may contribute to desensitization of KOR agonist responses (Nakagawa et al., 2001). Downstream of KOR, RGS12 attenuates G-protein signaling and promotes β-arrestin (Gross et al., 2019). Since β-arrestin is thought to promote unwanted effects of KOR agonists, including aversion, an inhibitor of RGS12 would be expected to promote G-protein signaling and therefore analgesia without dysphoria, as indicated in Figure 1.

### The Nociceptin Receptor

The genetic loci for the nociceptin (NOP) receptor and RGS19 neighbor each other, with RGS19 found only 83 base pairs from the 5′ end of the gene encoding NOP receptor (Ito et al., 2000; Xie et al., 2003). This 83 base pair region functions as a bidirectional promoter for both genes (Ito et al., 2000). Despite this close co-regulation RGS19 and NOP receptor expression show differences, for example, RGS19 is found in both undifferentiated and differentiated NT2 cells, while the NOP receptor is expressed only after differentiation (Ito et al., 2000). Nociceptin has both pro- and anti-nociceptive activity (Rizzi et al., 2016), so it will be of interest to see how the balance of these activities is controlled by members of the RGS protein family.

## Can We Target RGS Proteins?

An extensive amount of research has been conducted at both the molecular/cellular and behavioral levels on the interaction between MOR and certain RGS proteins, especially RGS4 and RGS9-2, but effects of RGS proteins on other opioid receptors is in its infancy. Nonetheless, the findings summarized above suggest that RGS proteins are attractive targets that may allow more precise control of opioid analgesic effects, and RGS inhibiting molecules may even have stand-alone analgesic efficacy.

In this regard the first attempts to develop inhibitors were peptides designed on the Gα interface with RGS proteins (Roof et al., 2008). To date, no small molecules targeting this large surface region have been published. On the other hand, a number of small molecules that act at a least one cysteine distant from the interaction surface have been developed (Roman et al., 2007, 2009; Blazer et al., 2010, 2011; Monroy et al., 2013; Storaska et al., 2013). Inhibition occurs by covalent modification of this cysteine, although the interaction of some inhibitors with RGS protein can be reversed (Storaska et al., 2013). Because many RGS proteins have a cysteine in the RH domain these inhibitors act somewhat promiscuously, although some degree of selectivity can be obtained. For example, CCG-203769 which inhibits RGS4, is 10-fold less potent at inhibiting RGS19, but has a very high selectivity over other members of the RGS family (Blazer et al., 2015). Similarly, CCG-50014 inhibits both RGS4 and RGS19 and is selective for these RGS proteins over RGS8 and RGS16 (Blazer et al., 2011), None of the small molecule inhibitors identified to date act at RGS6 or RGS7 (Hayes et al., 2018) which lack a cysteine in the RH domain, although it is worth noting that RGS9 does have a cysteine in the same position at the sensitive cysteine in RGS19. Of interest is that studies have now shown CCG-50014 also inhibits RGS1,5,14, and 17 and in fact is most potent at RGS 14 (Hayes et al., 2018). This could be significant given the, albeit limited, knowledge on the role of RGS14 in morphine antinociception (Garzón et al., 2001; Rodríguez-Muñoz et al., 2007a). Thus the finding that CCG-50014 enhances the inhibitory effects of both MOR and DOR agonists *in vitro* (Blazer et al., 2015), and, as mentioned earlier produces naloxone-reversible antinociception in a mouse model (Yoon et al., 2015), may not be due only to its action as an inhibitor of RGS4.

A report by Shaw et al. (2018), explores the structural determinants of RGS inhibitor selectivity. In general, inhibitors such as CCG-50014 preferentially inhibit RGS proteins with a greater degree of structural flexibility. Thus, increasing the number of interhelical salt bridges present in the RGS protein structure reduces flexibility, and decreases the relative affinity of CCG-50014 for RGS4 or RGS8. Conversely, mutations which decrease the rigidity of RGS4 and RGS8 increase CCG-50014 binding to these targets. In addition, a distinct class of small molecules, BMS-195270 and BMS-192364 were identified in an *in vitro* assay for bladder contraction using a chemical genetics screen. These compounds gave results consistent with a mechanism whereby they interfere with the Gα_*q*_/RGS complex downstream of muscarinic receptors to terminate signaling (Fitzgerald et al., 2006). Compounds with activity at Gα_i/o_/RGS complexes that are likely to be effective downstream of opioid receptors have not been described to our knowledge.

Overall, there is evidence for the involvement of certain RGS proteins in the control of pain and analgesia, although many of these studies measured only acute antinociception and have not been replicated or followed up. Moreover, the ability to selectively target these proteins, especially with reversible ligands is also very limited. As understanding of the binding modes for current RGS inhibitors continues to increase and new inhibitors are discovered, a more thorough understanding of the role of RGS proteins in pain and analgesia will become increasingly important.

## Author Contributions

NS wrote the original draft, and reviewed and corrected the manuscript. RK reviewed and revised the manuscript. KK prepared the figures, and reviewed and revised the manuscript. JT conceptualized the review, finalized the manuscript, and provided funding. All authors read and approved the submitted version.

## Conflict of Interest

The authors declare that the research was conducted in the absence of any commercial or financial relationships that could be construed as a potential conflict of interest.

## References

[B1] Ahlers K. E. Chakravarti B. Fisher R. A. (2016). RGS6 as a novel therapeutic target in CNS diseases and cancer. *AAPS J.* 18 560–572. 10.1208/s12248-016-9899-9 27002730 PMC5256616

[B2] Avrampou K. Pryce K. D. Ramakrishnan A. Sakloth F. Gaspari S. Serafini R. A. (2019). RGS4 maintains chronic pain symptoms in rodent models. *J. Neurosci.* 39 8291–8304. 10.1523/JNEUROSCI.3154-18.2019 31308097 PMC6794935

[B3] Babu K. M. Brent J. Juurlink D. N. (2019). Prevention of opioid overdose. *New Engl. J. Med.* 380 2246–2255. 10.1056/NEJMra1807054 31167053

[B4] Bertorelli R. Corradini L. Rafiq K. Tupper J. Caló G. Ongini E. (1999). Nociceptin and the ORL-1 ligand [Phe^1^ψ (CH_2_-NH)Gly^2^]nociceptin(1-13)NH_2_ exert anti-opioid effects in the Freund’s adjuvant-induced arthritic rat model of chronic pain. *Br. J. Pharmacol.* 128 1252–1258. 10.1038/sj.bjp.0702884 10578139 PMC1571738

[B5] Blazer L. L. Roman D. L. Chung A. Larsen M. J. Greedy B. M. Husbands S. M. (2010). Reversible, allosteric small-molecule inhibitors of regulator of G protein signaling proteins. *Mol. Pharmacol.* 78 524–533. 10.1124/mol.110.065128 20571077 PMC2939488

[B6] Blazer L. L. Storaska A. J. Jutkiewicz E. M. Turner E. M. Calcagno M. Wade S. M. (2015). Selectivity and anti-Parkinson’s potential of thiadiazolidinone RGS4 inhibitors. *ACS Chem. Neurosci.* 6 911–919. 10.1021/acschemneuro.5b00063 25844489

[B7] Blazer L. L. Zhang H. Casey E. M. Husbands S. M. Neubig R. R. (2011). A nanomolar-potency small molecule inhibitor of regulator of G-protein signaling proteins. *Biochemistry* 50 3181–3192. 10.1021/bi1019622 21329361 PMC3090679

[B8] Bosier B. Doyen P. J. Brolet A. Muccioli G. G. Ahmed E. Desmet N. (2015). Inhibition of the regulator of G protein signalling RGS4 in the spinal cord decreases neuropathic hyperalgesia and restores cannabinoid CB1 receptor signalling. *Br. J. Pharmacol.* 172 5333–5346. 10.1111/bph.13324 26478461 PMC5341217

[B9] Burford N. T. Clark M. J. Wehrman T. S. Gerritz S. W. Banks M. O’Connell J. (2013). Discovery of positive allosteric modulators and silent allosteric modulators of the μ-opioid receptor. *Proc. Natl. Acad. Sci. U.S.A.* 110 10830–10835. 10.1073/pnas.1300393110 23754417 PMC3696790

[B10] Burford N. T. Traynor J. R. Alt A. (2015). Positive allosteric modulators of the μ-opioid receptor: a novel approach for future pain medications. *Br. J. Pharmacol.* 172 277–286. 10.1111/bph.12599 24460691 PMC4292945

[B11] Cesselin F. Montastruc J. L. Gros C. Bourgoin S. Hamon M. (1980). Met-enkephalin levels and opiate receptors in the spinal cord of chronic suffering rats. *Brain Res.* 191 289–293. 10.1016/0006-8993(80)90335-2 6247014

[B12] Clark M. J. Harrison C. Zhong H. Neubig R. R. Traynor J. R. (2003). Endogenous RGS protein action modulates μ-opioid signaling through Gα_o_. Effects on adenylyl cyclase, extracellular signal-regulated kinases, and intracellular calcium pathways. *J. Biol. Chem.* 278 9418–9425. 10.1074/jbc.M208885200 12524446

[B13] Costigan M. Samad T. A. Allchorne A. Lanoue C. Tate S. Woolf C. J. (2003). High basal expression and injury-induced down regulation of two regulator of G-protein signaling transcripts. RGS3 and RGS4 in primary sensory neurons. *Mol. Cell. Neurosci.* 24 106–116. 10.1016/S1044-7431(03)00135-0 14550772

[B14] Doupnik C. A. (2015). RGS redundancy and implications in GPCR-GIRK signaling. *Int. Rev. Neurobiol.* 123 87–116. 10.1016/bs.irn.2015.05.010 26422983

[B15] Doyen P. J. Vergouts M. Pochet A. Desmet N. van Neerven S. Brook G. (2017). Inflammation-associated regulation of RGS in astrocytes and putative implication in neuropathic pain. *J. Neuroinflam.* 14:209. 10.1186/s12974-017-0971-x 29078779 PMC5658970

[B16] Dripps I. J. Wang Q. Neubig R. R. Rice K. C. Traynor J. R. Jutkiewicz E. M. (2017). The role of regulator of G protein signaling 4 in delta-opioid receptor-mediated behaviors. *Psychopharmacology* 234 29–39. 10.1007/s00213-016-4432-5 27624599 PMC5203942

[B17] Elenko E. Fischer T. Niesman I. Harding T. McQuistan T. Von Zastrow M. (2003). Spatial regulation of Gαi protein signaling in clathrin-coated membrane microdomains containing GAIP. *Mol. Pharmacol.* 64 11–20. 10.1124/mol.64.1.11 12815156

[B18] Fitzgerald K. Tertyshnikova S. Moore L. Bjerke L. Burley B. Cao J. (2006). Chemical genetics reveals an RGS/G-protein role in the action of a compound. *PLoS Genet.* 2:e57. 10.1371/journal.pgen.0020057 16683034 PMC1440875

[B19] Fournié-Zaluski M. C. Coric P. Turcaud S. Lucas E. Noble F. Maldonado R. (1992). “Mixed inhibitor-prodrug” as a new approach toward systemically active inhibitors of enkephalin-degrading enzymes. *J. Med. Chem.* 35 2473–2481. 10.1021/jm00091a016 1352352

[B20] Garnier M. Zaratin P. F. Ficalora G. Valente M. Fontanella L. Rhee M.-H. (2003). Up-regulation of regulator of G protein signaling 4 expression in a model of neuropathic pain and insensitivity to morphine. *J. Pharmacol. Exp. Ther.* 304 1299–1306. 10.1124/jpet.102.043471 12604710

[B21] Garzón J. de Antonio I. Sánchez-Blázquez P. (2000). In vivo modulation of G proteins and opioid receptor function by antisense oligodeoxynucleotides. *Methods Enzymol.* 314 3–20. 10.1016/s0076-6879(99)14091-6 10565001

[B22] Garzón J. López-Fando A. Sánchez-Blázquez P. (2003). The R7 subfamily of RGS proteins assists tachyphylaxis and acute tolerance at mu-opioid receptors. *Neuropsychopharmacology* 28 1983–1990. 10.1038/sj.npp.1300263 12902995

[B23] Garzón J. Rodríguez-Díaz M. López-Fando A. Sánchez-Blázquez P. (2001). RGS9 proteins facilitate acute tolerance to mu-opioid effects. *Eur. J. Neurosci.* 13 801–811. 10.1046/j.0953-816x.2000.01444.x 11207815

[B24] Garzón J. Rodríguez-Muñoz M. López-Fando A. García-España A. Sánchez-Blázquez P. (2004). RGSZ1 and GAIP regulate mu- but not delta-opioid receptors in mouse CNS: role in tachyphylaxis and acute tolerance. *Neuropsychopharmacology* 29 1091–1104. 10.1038/sj.npp.1300408 14997173

[B25] Garzón J. Rodríguez-Muñoz M. López-Fando A. Sánchez-Blázquez P. (2005). The RGSZ2 protein exists in a complex with mu-opioid receptors and regulates the desensitizing capacity of Gz proteins. *Neuropsychopharmacology* 30 1632–1648. 10.1038/sj.npp.1300726 15827571

[B26] Gaspari S. Cogliani V. Manouras L. Anderson E. M. Mitsi V. Avrampou K. (2017). RGS9-2 modulates responses to oxycodone in pain-free and chronic pain states. *Neuropsychopharmacology* 42 1548–1556. 10.1038/npp.2017.4 28074831 PMC5436127

[B27] Gaspari S. Purushothaman I. Cogliani V. Sakloth F. Neve R. L. Howland D. (2018). Suppression of RGSz1 function optimizes the actions of opioid analgesics by mechanisms that involve the Wnt/β-catenin pathway. *Proc. Natl. Acad. Sci. U.S. A.* 115 E2085–E2094. 10.1073/pnas.1707887115 29440403 PMC5834666

[B28] Gold S. J. Han M.-H. Herman A. E. Ni Y. G. Pudiak C. M. Aghajanian G. K. (2003). Regulation of RGS proteins by chronic morphine in rat locus coeruleus. *Eur. J. Neurosci.* 17 971–980. 10.1046/j.1460-9568.2003.02529.x 12653973

[B29] Gold S. J. Ni Y. G. Dohlman H. G. Nestler E. J. (1997). Regulators of G-protein signaling (RGS) proteins: region-specific expression of nine subtypes in rat brain. *J. Neurosci.* 17 8024–8037. 10.1523/jneurosci.17-20-08024.1997 9315921 PMC6793903

[B30] Grafstein-Dunn E. Young K. H. Cockett M. I. Khawaja X. Z. (2001). Regional distribution of regulators of G-protein signaling (RGS) 1, 2, 13, 14, 16, and GAIP messenger ribonucleic acids by in situ hybridization in rat brain. *Brain Res.* 88 113–123. 10.1016/s0169-328x(01)00038-9 11295237

[B31] Grillet N. Dubreuil V. Dufour H. D. Brunet J.-F. (2003). Dynamic expression of RGS4 in the developing nervous system and regulation by the neural type-specific transcription factor Phox2b. *J. Neurosci.* 23 10613–10621. 10.1523/JNEUROSCI.23-33-10613.2003 14627646 PMC6740911

[B32] Grillet N. Pattyn A. Contet C. Kieffer B. L. Goridis C. Brunet J.-F. (2005). Generation and characterization of RGS4 mutant mice. *Mol. Cell. Biol.* 25 4221–4228. 10.1128/MCB.25.10.4221-4228.2005 15870291 PMC1087729

[B33] Gross J. D. Kaski S. W. Schmidt K. T. Cogan E. S. Boyt K. M. Wix K. (2019). Role of RGS12 in the differential regulation of kappa opioid receptor-dependent signaling and behavior. *Neuropsychopharmacology* 44 1728–1741. 10.1038/s41386-019-0423-7 31141817 PMC6785087

[B34] Han M.-H. Renthal W. Ring R. H. Rahman Z. Psifogeorgou K. Howland D. (2010). Brain region specific actions of regulator of G protein signaling 4 oppose morphine reward and dependence but promote analgesia. *Biol. Psychiatry* 67 761–769. 10.1016/j.biopsych.2009.08.041 19914603 PMC3077672

[B35] Hayes M. P. Bodle C. R. Roman D. L. (2018). Evaluation of the selectivity and cysteine dependence of inhibitors across the regulator of G protein-signaling family. *Mol. Pharmacol.* 93 25–35. 10.1124/mol.117.109843 29051318 PMC5708088

[B36] Hepler J. R. Berman D. M. Gilman A. G. Kozasa T. (1997). RGS4 and GAIP are GTPase-activating proteins for Gq alpha and block activation of phospholipase C beta by gamma-thio-GTP-Gq alpha. *Proc. Natl. Acad. Sci. U.S.A.* 94 428–432. 10.1073/pnas.94.2.428 9012799 PMC19528

[B37] Hollinger S. Hepler J. R. (2002). Cellular regulation of RGS proteins: modulators and integrators of G protein signaling. *Pharmacol. Rev.* 54 527–559. 10.1124/pr.54.3.527 12223533

[B38] Hurley R. W. Hammond D. L. (2001). Contribution of endogenous enkephalins to the enhanced analgesic effects of supraspinal mu opioid receptor agonists after inflammatory injury. *J. Neurosci.* 21 2536–2545. 10.1523/JNEUROSCI.21-07-02536.2001 11264327 PMC6762402

[B39] Ito E. Xie G. Maruyama K. Palmer P. P. (2000). A core-promoter region functions bi-directionally for human opioid-receptor-like gene ORL1 and its 5’-adjacent gene GAIP. *J. Mol. Biol.* 304 259–270. 10.1006/jmbi.2000.4212 11090272

[B40] Karoussiotis C. Marti-Solano M. Stepniewski T. M. Symeonof A. Selent J. Georgoussi Z. (2019). A highly conserved δ-opioid receptor region determines RGS4 interaction. *FEBS J.* 10.1111/febs.15033 31386272

[B41] Khroyan T. V. Polgar W. E. Jiang F. Zaveri N. T. Toll L. (2009). Nociceptin/orphanin FQ receptor activation attenuates antinociception induced by mixed nociceptin/orphanin FQ/μ-opioid receptor agonists. *J. Pharmacol. Exp. Ther.* 331 946–953. 10.1124/jpet.109.156711 19713488 PMC2784721

[B42] Kroenke K. Krebs E. E. Bair M. J. (2009). Pharmacotherapy of chronic pain: a synthesis of recommendations from systematic reviews. *Gen. Hospital Psychiatry* 31 206–219. 10.1016/j.genhosppsych.2008.12.006 19410099

[B43] Lamberts J. T. Jutkiewicz E. M. Mortensen R. M. Traynor J. R. (2011). μ-Opioid receptor coupling to Gα(o) plays an important role in opioid antinociception. *Neuropsychopharmacology* 36 2041–2053. 10.1038/npp.2011.91 21654736 PMC3158321

[B44] Lamberts J. T. Smith C. E. Li M.-H. Ingram S. L. Neubig R. R. Traynor J. R. (2013). Differential control of opioid antinociception to thermal stimuli in a knock-in mouse expressing regulator of G-protein signaling-insensitive Gαo protein. *J. Neurosci.* 33 4369–4377. 10.1523/JNEUROSCI.5470-12.2013 23467353 PMC3740968

[B45] Lamberts J. T. Traynor J. R. (2013). Opioid receptor interacting proteins and the control of opioid signaling. *Curr. Pharmaceut. Design* 19 7333–7347. 10.2174/138161281942140105160625 23448476 PMC6707067

[B46] Lan K. L. Zhong H. Nanamori M. Neubig R. R. (2000). Rapid kinetics of regulator of G-protein signaling (RGS)-mediated Galphai and Galphao deactivation. Galpha specificity of RGS4 AND RGS7. *J. Biol. Chem.* 275 33497–33503. 10.1074/jbc.M005785200 10942773

[B47] Leontiadis L. J. Papakonstantinou M. P. Georgoussi Z. (2009). Regulator of G protein signaling 4 confers selectivity to specific G proteins to modulate mu- and delta-opioid receptor signaling. *Cell. Sign.* 21 1218–1228. 10.1016/j.cellsig.2009.03.013 19324084

[B48] Levine J. D. Gordon N. C. Jones R. T. Fields H. L. (1978). The narcotic antagonist naloxone enhances clinical pain. *Nature* 272 826–827. 10.1038/272826a0 347307

[B49] Livingston K. E. Traynor J. R. (2018). Allostery at opioid receptors: modulation with small molecule ligands. *Br. J. Pharmacol.* 175 2846–2856. 10.1111/bph.13823 28419415 PMC6016636

[B50] Manglik A. Lin H. Aryal D. K. McCorvy J. D. Dengler D. Corder G. (2016). Structure-based discovery of opioid analgesics with reduced side effects. *Nature* 537 185–190. 10.1038/nature19112 27533032 PMC5161585

[B51] Markman J. Gudin J. Rauck R. Argoff C. Rowbotham M. Agaiby E. (2019). SUMMIT-07: a randomized trial of NKTR-181, a new molecular entity, full mu-opioid receptor agonist for chronic low-back pain. *Pain* 160 1374–1382. 10.1097/j.pain.0000000000001517 30747908 PMC6553961

[B52] McPherson K. B. Leff E. R. Li M.-H. Meurice C. Tai S. Traynor J. R. (2018). Regulators of G-protein signaling (RGS) proteins promote receptor coupling to g-protein-coupled inwardly rectifying potassium (GIRK) channels. *J. Neurosci.* 38 8737–8744. 10.1523/JNEUROSCI.0516-18.2018 30150362 PMC6181307

[B53] Monroy C. A. Doorn J. A. Roman D. L. (2013). Modification and functional inhibition of regulator of G-protein signaling 4 (RGS4) by 4-hydroxy-2-nonenal. *Chem. Res. Toxicol.* 26 1832–1839. 10.1021/tx400212q 24229325 PMC4441825

[B54] Nakagawa T. Minami M. Satoh M. (2001). Up-regulation of RGS4 mRNA by opioid receptor agonists in PC12 cells expressing cloned mu- or kappa-opioid receptors. *Eur. J. Pharmacol.* 433 29–36. 10.1016/s0014-2999(01)01485-6 11755131

[B55] Nastase A. F. Griggs N. W. Anand J. P. Fernandez T. J. Harland A. A. Trask T. J. (2018). Synthesis and pharmacological evaluation of novel C-8 substituted tetrahydroquinolines as balanced-affinity Mu/Delta opioid ligands for the treatment of pain. *ACS Chem. Neurosci.* 9 1840–1848. 10.1021/acschemneuro.8b00139 29677442 PMC9976708

[B56] Ni Y. G. Gold S. J. Iredale P. A. Terwilliger R. Z. Duman R. S. Nestler E. J. (1999). Region-specific regulation of RGS4 (Regulator of G-protein-signaling protein type 4) in brain by stress and glucocorticoids: in vivo and in vitro studies. *J. Neurosci.* 19 3674–3680. 10.1523/JNEUROSCI.19-10-03674.19992002 10233999 PMC6782705

[B57] Noble F. Smadja C. Valverde O. Maldonado R. Coric P. Turcaud S. (1997). Pain-suppressive effects on various nociceptive stimuli (thermal, chemical, electrical and inflammatory) of the first orally active enkephalin-metabolizing enzyme inhibitor RB 120. *Pain* 73 383–391. 10.1016/s0304-3959(97)00125-5 9469529

[B58] Noble F. Soleilhac J. M. Soroca-Lucas E. Turcaud S. Fournie-Zaluski M. C. Roques B. P. (1992). Inhibition of the enkephalin-metabolizing enzymes by the first systemically active mixed inhibitor prodrug RB 101 induces potent analgesic responses in mice and rats. *J. Pharmacol. Exp. Ther.* 261 181–190. 1560364

[B59] Papachatzaki M. M. Antal Z. Terzi D. Szücs P. Zachariou V. Antal M. (2011). RGS9-2 modulates nociceptive behaviour and opioid-mediated synaptic transmission in the spinal dorsal horn. *Neurosci. Lett.* 501 31–34. 10.1016/j.neulet.2011.06.033 21741448 PMC3394093

[B60] Papakonstantinou M.-P. Karoussiotis C. Georgoussi Z. (2015). RGS2 and RGS4 proteins: new modulators of the κ-opioid receptor signaling. *Cell. Sign.* 27 104–114. 10.1016/j.cellsig.2014.09.023 25289860

[B61] Peckys D. Landwehrmeyer G. B. (1999). Expression of mu, kappa, and delta opioid receptor messenger RNA in the human CNS: a 33P in situ hybridization study. *Neuroscience* 88 1093–1135. 10.1016/s0306-4522(98)00251-6 10336124

[B62] Porro C. A. Tassinari G. Facchinetti F. Panerai A. E. Carli G. (1991). Central beta-endorphin system involvement in the reaction to acute tonic pain. *Exp. Brain Res.* 83 549–554. 10.1007/bf00229833 2026197

[B63] Posner B. A. Gilman A. G. Harris B. A. (1999). Regulators of G protein signaling 6 and 7. Purification of complexes with gbeta5 and assessment of their effects on g protein-mediated signaling pathways. *J. Biol. Chem.* 274 31087–31093. 10.1074/jbc.274.43.31087 10521509

[B64] Psifogeorgou K. Papakosta P. Russo S. J. Neve R. L. Kardassis D. Gold S. J. (2007). RGS9-2 is a negative modulator of mu-opioid receptor function. *J. Neurochem.* 103 617–625. 10.1111/j.1471-4159.2007.04812.x 17725581

[B65] Psifogeorgou K. Psigfogeorgou K. Terzi D. Papachatzaki M. M. Varidaki A. Ferguson D. (2011). A unique role of RGS9-2 in the striatum as a positive or negative regulator of opiate analgesia. *J. Neurosci.* 31 5617–5624. 10.1523/JNEUROSCI.4146-10.2011 21490202 PMC3412365

[B66] Raehal K. M. Schmid C. L. Groer C. E. Bohn L. M. (2011). Functional selectivity at the μ-opioid receptor: implications for understanding opioid analgesia and tolerance. *Pharmacol. Rev.* 63 1001–1019. 10.1124/pr.111.004598 21873412 PMC3186080

[B67] Rizzi A. Cerlesi M. C. Ruzza C. Malfacini D. Ferrari F. Bianco S. (2016). Pharmacological characterization of cebranopadol a novel analgesic acting as mixed nociceptin/orphanin FQ and opioid receptor agonist. *Pharmacol. Res. Persp.* 4:e00247. 10.1002/prp2.247 28116100 PMC5242173

[B68] Rodríguez-Muñoz M. de la Torre-Madrid E. Gaitán G. Sánchez-Blázquez P. Garzón J. (2007a). RGS14 prevents morphine from internalizing Mu-opioid receptors in periaqueductal gray neurons. *Cell. Sign.* 19 2558–2571. 10.1016/j.cellsig.2007.08.003 17825524

[B69] Rodríguez-Muñoz M. de la Torre-Madrid E. Sánchez-Blázquez P. Garzón J. (2007b). Morphine induces endocytosis of neuronal mu-opioid receptors through the sustained transfer of Galpha subunits to RGSZ2 proteins. *Mol. Pain* 3:19. 10.1186/1744-8069-3-19 17634133 PMC1947952

[B70] Roman D. L. Ota S. Neubig R. R. (2009). Polyplexed flow cytometry protein interaction assay: a novel high-throughput screening paradigm for RGS protein inhibitors. *J. Biomol. Screen.* 14 610–619. 10.1177/1087057109336590 19531661 PMC2908316

[B71] Roman D. L. Talbot J. N. Roof R. A. Sunahara R. K. Traynor J. R. Neubig R. R. (2007). Identification of small-molecule inhibitors of RGS4 using a high-throughput flow cytometry protein interaction assay. *Mol. Pharmacol.* 71 169–175. 10.1124/mol.106.028670 17012620

[B72] Roof R. A. Sobczyk-Kojiro K. Turbiak A. J. Roman D. L. Pogozheva I. D. Blazer L. L. (2008). Novel peptide ligands of RGS4 from a focused one-bead, one-compound library. *Chem. Biol. Drug Design* 72 111–119. 10.1111/j.1747-0285.2008.00687.x 18637987 PMC2917810

[B73] Roy A. A. Lemberg K. E. Chidiac P. (2003). Recruitment of RGS2 and RGS4 to the plasma membrane by G proteins and receptors reflects functional interactions. *Mol. Pharmacol.* 64 587–593. 10.1124/mol.64.3.587 12920194

[B74] Sánchez-Blázquez P. Rodríguez-Díaz M. López-Fando A. Rodríguez-Muñoz M. Garzón J. (2003). The GBeta5 subunit that associates with the R7 subfamily of RGS proteins regulates mu-opioid effects. *Neuropharmacology* 45 82–95. 10.1016/s0028-3908(03)00149-7 12814661

[B75] Schmid C. L. Kennedy N. M. Ross N. C. Lovell K. M. Yue Z. Morgenweck J. (2017). Bias factor and therapeutic window correlate to predict safer opioid analgesics. *Cell* 171:1165-1175.e18. 10.1016/j.cell.2017.10.035 29149605 PMC5731250

[B76] Shaw V. S. Mohammadiarani H. Vashisth H. Neubig R. R. (2018). Differential protein dynamics of regulators of G-protein signaling: role in specificity of small-molecule inhibitors. *J. Am. Chem. Soc.* 140 3454–3460. 10.1021/jacs.7b13778 29460621 PMC6309336

[B77] Sierra D. A. Gilbert D. J. Householder D. Grishin N. V. Yu K. Ukidwe P. (2002). Evolution of the regulators of G-protein signaling multigene family in mouse and human. *Genomics* 79 177–185. 10.1006/geno.2002.6693 11829488

[B78] Stein C. Schäfer M. Machelska H. (2003). Attacking pain at its source: new perspectives on opioids. *Nat. Med.* 9 1003–1008. 10.1038/nm908 12894165

[B79] Storaska A. J. Mei J. P. Wu M. Li M. Wade S. M. Blazer L. L. (2013). Reversible inhibitors of regulators of G-protein signaling identified in a high-throughput cell-based calcium signaling assay. *Cell. Sign.* 25 2848–2855. 10.1016/j.cellsig.2013.09.007 24041654 PMC3848259

[B80] Stratinaki M. Varidaki A. Mitsi V. Ghose S. Magida J. Dias C. (2013). Regulator of G protein signaling 4 [corrected] is a crucial modulator of antidepressant drug action in depression and neuropathic pain models. *Proc. Natl. Acad. Sci. U.S.A.* 110 8254–8259. 10.1073/pnas.1214696110 23630294 PMC3657820

[B81] Taccola G. Doyen P. J. Damblon J. Dingu N. Ballarin B. Steyaert A. (2016). A new model of nerve injury in the rat reveals a role of regulator of G protein signaling 4 in tactile hypersensitivity. *Exp. Neurol.* 286 1–11. 10.1016/j.expneurol.2016.09.008 27641322

[B82] Talbot J. N. Roman D. L. Clark M. J. Roof R. A. Tesmer J. J. G. Neubig R. R. (2010). Differential modulation of mu-opioid receptor signaling to adenylyl cyclase by regulators of G protein signaling proteins 4 or 8 and 7 in permeabilised C6 cells is Galpha subtype dependent. *J. Neurochem.* 112 1026–1034. 10.1111/j.1471-4159.2009.06519.x 20002516 PMC2947325

[B83] Terzi D. Cao Y. Agrimaki I. Martemyanov K. A. Zachariou V. (2012). R7BP modulates opiate analgesia and tolerance but not withdrawal. *Neuropsychopharmacology* 37 1005–1012. 10.1038/npp.2011.284 22089315 PMC3280654

[B84] Terzi D. Gaspari S. Manouras L. Descalzi G. Mitsi V. Zachariou V. (2014). RGS9-2 modulates sensory and mood related symptoms of neuropathic pain. *Neurobiol. Learn. Mem.* 115 43–48. 10.1016/j.nlm.2014.08.005 25150149 PMC4459754

[B85] Terzi D. Stergiou E. King S. L. Zachariou V. (2009). Regulators of G protein signaling in neuropsychiatric disorders. *Prog. Mol. Biol. Transl. Sci.* 86 299–333. 10.1016/S1877-1173(09)86010-9 20374720

[B86] Tesmer J. J. Berman D. M. Gilman A. G. Sprang S. R. (1997). Structure of RGS4 bound to AlF4–activated G(i alpha1): Stabilization of the transition state for GTP hydrolysis. *Cell* 89 251–261. 10.1016/s0092-8674(00)80204-4 9108480

[B87] Traynor J. R. Neubig R. R. (2005). Regulators of G protein signaling & drugs of abuse. *Mol. Interv.* 5 30–41. 10.1124/mi.5.1.7 15734717

[B88] Ulens C. Daenens P. Tytgat J. (2000). Changes in GIRK1/GIRK2 deactivation kinetics and basal activity in the presence and absence of RGS4. *Life Sci.* 67 2305–2317. 10.1016/s0024-3205(00)00820-1 11065178

[B89] Violin J. D. Crombie A. L. Soergel D. G. Lark M. W. (2014). Biased ligands at G-protein-coupled receptors: Promise and progress. *Trends Pharmacol. Sci.* 35 308–316. 10.1016/j.tips.2014.04.007 24878326

[B90] Wang Q. Liu-Chen L.-Y. Traynor J. R. (2009). Differential modulation of mu- and delta-opioid receptor agonists by endogenous RGS4 protein in SH-SY5Y cells. *J. Biol. Chem.* 284 18357–18367. 10.1074/jbc.M109.015453 19416973 PMC2709384

[B91] Wang Q. Traynor J. R. (2013). Modulation of μ-opioid receptor signaling by RGS19 in SH-SY5Y cells. *Mol. Pharmacol.* 83 512–520. 10.1124/mol.112.081992 23197645 PMC3558815

[B92] Wu H. Hung K. Ohsawa M. Mizoguchi H. Tseng L. F. (2001). Antisera against endogenous opioids increase the nocifensive response to formalin: demonstration of inhibitory beta-endorphinergic control. *Eur. J. Pharmacol.* 421 39–43. 10.1016/s0014-2999(01)00970-0 11408047

[B93] Xie G. Han X. Ito E. Yanagisawa Y. Maruyama K. Sugano S. (2003). Gene structure, dual-promoters and mRNA alternative splicing of the human and mouse regulator of G protein signaling GAIP/RGS19. *J. Mol. Biol.* 325 721–732. 10.1016/s0022-2836(02)01283-4 12507475

[B94] Xu X. Zeng W. Popov S. Berman D. M. Davignon I. Yu K. (1999). RGS proteins determine signaling specificity of Gq-coupled receptors. *J. Biol. Chem.* 274 3549–3556. 10.1074/jbc.274.6.3549 9920901

[B95] Yoon S.-Y. Woo J. Park J.-O. Choi E.-J. Shin H.-S. Roh D.-H. (2015). Intrathecal RGS4 inhibitor, CCG50014, reduces nociceptive responses and enhances opioid-mediated analgesic effects in the mouse formalin test. *Anesthesia Analg.* 120 671–677. 10.1213/ANE.0000000000000607 25695583

[B96] Zachariou V. Georgescu D. Sanchez N. Rahman Z. DiLeone R. Berton O. (2003). Essential role for RGS9 in opiate action. *Proc. Natl. Acad. Sci. U.S.A.* 100 13656–13661. 10.1073/pnas.2232594100 14595021 PMC263869

[B97] Zangen A. Herzberg U. Vogel Z. Yadid G. (1998). Nociceptive stimulus induces release of endogenous beta-endorphin in the rat brain. *Neuroscience* 85 659–662. 10.1016/s0306-4522(98)00050-5 9639262

[B98] Zeng W. Xu X. Popov S. Mukhopadhyay S. Chidiac P. Swistok J. (1998). The N-terminal domain of RGS4 confers receptor-selective inhibition of G protein signaling. *J. Biol. Chem.* 273 34687–34690. 10.1074/jbc.273.52.34687 9856989

[B99] Zhong H. Wade S. M. Woolf P. J. Linderman J. J. Traynor J. R. Neubig R. R. (2003). A spatial focusing model for G protein signals. Regulator of G protein signaling (RGS) protien-mediated kinetic scaffolding. *J. Biol. Chem.* 278 7278–7284. 10.1074/jbc.M208819200 12446706

[B100] Zhou H. Chisari M. Raehal K. M. Kaltenbronn K. M. Bohn L. M. Mennerick S. J. (2012). GIRK channel modulation by assembly with allosterically regulated RGS proteins. *Proc. Natl. Acad. Sci. U.S.A.* 109 19977–19982. 10.1073/pnas.1214337109 23169654 PMC3523866

